# Impacts of drought and elevated temperature on the seeds of malting barley

**DOI:** 10.3389/fpls.2022.1049323

**Published:** 2022-12-08

**Authors:** Manuela Nagel, Erwann Arc, Loïc Rajjou, Gwendal Cueff, Marlene Bailly, Gilles Clément, Inmaculada Sanchez-Vicente, Christophe Bailly, Charlotte E. Seal, Thomas Roach, Hardy Rolletschek, Oscar Lorenzo, Andreas Börner, Ilse Kranner

**Affiliations:** ^1^ Genebank Department, Leibniz Institute of Plant Genetics and Crop Plant Research (IPK), Gatersleben, Seeland, Germany; ^2^ Department of Botany and Center for Molecular Biosciences Innsbruck (CMBI), University of Innsbruck, Innsbruck, Austria; ^3^ Université Paris-Saclay, INRAE, AgroParisTech, Institut Jean-Pierre Bourgin (IJPB), Versailles, France; ^4^ Department of Botany and Plant Physiology, Instituto de Investigación en Agrobiotecnología (CIALE), Facultad de Biología, Universidad de Salamanca, Salamanca, Spain; ^5^ Unité Mixte de Recherche (UMR) 7622 Biologie du Développement, Institut de Biologie Paris Seine (IBPS), Sorbonne Université, CNRS, Paris, France; ^6^ Royal Botanic Gardens, Kew, Wakehurst, Ardingly, Haywards Heath, West Sussex, Haywards Heath, United Kingdom

**Keywords:** barley, climate change, dwarfing genes, drought, metabolite, temperature, seed viability and seedling growth, ABI5 (ABA insensitive 5) and ANAC089

## Abstract

High seed quality is key to agricultural production, which is increasingly affected by climate change. We studied the effects of drought and elevated temperature during seed production on key seed quality traits of two genotypes of malting barley (*Hordeum sativum* L.). Plants of a “Hana-type” landrace (B1) were taller, flowered earlier and produced heavier, larger and more vigorous seeds that resisted ageing longer compared to a semi-dwarf breeding line (B2). Accordingly, a NAC domain-containing transcription factor (TF) associated with rapid response to environmental stimuli, and the TF ABI5, a key regulator of seed dormancy and vigour, were more abundant in B1 seeds. Drought significantly reduced seed yield in both genotypes, and elevated temperature reduced seed size. Genotype B2 showed partial thermodormancy that was alleviated by drought and elevated temperature. Metabolite profiling revealed clear differences between the embryos of B1 and B2. Drought, but not elevated temperature, affected the metabolism of amino acids, organic acids, osmolytes and nitrogen assimilation, in the seeds of both genotypes. Our study may support future breeding efforts to produce new lodging and drought resistant malting barleys without trade-offs that can occur in semi-dwarf varieties such as lower stress resistance and higher dormancy.

## Introduction

Barley (*Hordeum vulgare* L.) is among the four most important crops globally (http://faostat.fao.org) and its caryopses, henceforth termed seeds, are used for staple food, for animal feed and in the brewing industry. Malting barley varieties must meet specific quality criteria, especially low lodging potential and high tolerance against diseases, high and stable seed yield, a seed size > 2.5 mm and for malting, the seed protein content should not exceed 12% (Federal Variety Office, 2021) as higher proteins contents interfere with the malting process (Jaeger et al., 2021). Significant breeding success with malting barleys was achieved around 1900 when Austrian breeders, including Tschermak-Seysenegg, used spring barleys from the Hana region of the Czech Republic (then Central Moravia) for hybridization. Due to their high malting quality, ‘Hana’-type barleys dominated markets until the 1940s (Grausgruber et al., 2002). Later, i.e. during the Green Revolution, ‘Hana’-type barleys were largely replaced by semi-dwarf varieties that were less susceptible to lodging. Notably, up to a 4.7 fold increase in seed yield could be achieved using dwarf and semi-dwarf varieties (Jing and Wanxia, 2003), resulting in improved harvest index, and it was easier to control nitrogen supply to achieve optimal seed nitrogen contents (Laidig et al., 2017), whereas old landraces with longer stems are prone to lodging as they produce heavier spikes when receiving more nitrogen (Grausgruber et al., 2002). The shorter plant height of semi-dwarfs is due to short culm mutation alleles related to changes in the metabolism and signal transduction of gibberellins (Gao and Chu, 2020) or brassinosteroids (Dockter et al., 2014). Most modern barley varieties carry a mutation at the *semi-dwarf* (*sdw*) gene, such as the *Sdw1/Denso* gene (Kuczyńska et al., 2013; Dockter and Hansson, 2015), an orthologue of the rice Green Revolution gene *SD1* encoding gibberellin 20-oxidase *HvGA20ox2*, a 2-oxoglutarate-dependent dioxygenase involved in the last steps of GA synthesis (Xu et al., 2017). Modifications of biosynthesis and signal transduction pathways of these hormones were the basis for plant breeding progress, and thus for improved seed and malt quality of spring barley (Dockter et al., 2014; Dockter and Hansson, 2015).

A deterioration of agro-climatic conditions affecting cereal productivity is forecast for many areas globally (Zhao et al., 2017; Ortiz-Bobea et al., 2021). In Europe, more erratic precipitation and shortening of the active growing season between cold winters and hot summers (Trnka et al., 2014) have been shown to negatively affect spring barley yield (Gammans et al., 2017; Jacott and Boden, 2020) and malt quality (Howard et al., 1996). Specifically, drought during seed production increases barley seed protein contents and thus, impairs malt quality (Jaeger et al., 2021). Furthermore, in warmer and drier regions, undesirable higher protein contents with the associated lower malt extract were observed after nitrogen application (Eagles et al., 1995). However, for a comprehensive understanding of the effects of climate change on plant productivity, the effects of the environment on plant and seed physiology must be appreciated. Moreover, focus must not only be placed on the nutritional quality and industrially desirable traits of barley seeds, such as malt quality, but also on key seed quality traits that are the basis for crop production, such as seed germination, vigour, viability and dormancy. Seed germination, the first critical step in the life cycle of most crop plants, can be defined as the process by which a quiescent seed develops into a seedling, starting with water uptake and ending with the protrusion of the radicle (the embryonic root) through the surrounding covers in conjunction with cell division in the radicle (Bewley, 1997; Nonogaki, 2014). Seed vigour, often best observed under stress conditions, can be defined as the sum total of those properties, including germination speed, that determine seed performance during germination and seedling emergence (Finch-Savage and Bassel, 2015). Viability is the capability of a seed to germinate, and dormancy is the inability of a seed to germinate under favourable conditions before certain environmental cues, such as low temperatures for an extended time, have been received (Hampton and TeKrony, 1995; Graeber et al., 2012). Lack of dormancy may result in pre-harvest sprouting, i.e. germination on the mother plant, which is an undesirable agronomic trait that compromises yield, nutritional and processing quality, and in barley, renders seeds unsuitable for malting (Rodriguez et al., 2015). The depth of seed dormancy is determined during seed maturation on the mother plant, subject to environmental and parental control, through genetic and hormonal regulation (Penfield and MacGregor, 2017; Iwasaki et al., 2022).

Here, we report on the response of two malting barleys, the Austrian landrace HOR 2110 (B1), in the ‘Hana’ progeny, and a semi-dwarf breeding line HOR 4710 (B2) used in Northern Germany. These two genotypes were part of the “EcoSeed” mapping panel, which revealed that plant height, dormancy, number of seeds per spike and days to anthesis are heritable phenotypic traits (Nagel et al., 2019). We hypothesised that drought and elevated temperature during seed production differentially affect seed quality traits such as seed vigour, germination, dormancy and reserve deposition in the two genotypes. To assess seed stress level, responses of malondialdehyde (MDA), a marker of oxidative stress (Kranner et al., 2010), and two transcription factors (TFs), ABI5 and ANAC089, with prominent roles in the regulation of seed germination were studied. The TF ABI5, a member of the basic leucine zipper (bZIP) family, represents a hub in the abscisic acid (ABA) signalling network (Sanchez-Vicente et al., 2019) during seed germination and post-germinative developmental checkpoints (Finkelstein and Lynch, 2000; Lopez-Molina et al., 2001). In barley seeds, the ABI5 protein is expressed in the aleurone cells (Casaretto and Ho, 2003). Moreover, ABI5 is involved in the perception of various developmental cues and in stress response (Jakoby et al., 2002) and transgenic cotton expressing *AtABI5* showed enhanced resistance to drought stress (Mittal et al., 2014). ABI5 protein accumulation is triggered by ABA-induced phosphorylation resulting in the stabilization of the protein (Lopez-Molina et al., 2001). Furthermore, *S*-nitrosylation of ABI5 by nitric oxide (NO) leads to its proteolysis, thereby alleviating the repression of seed germination by ABI5 (Albertos et al., 2021). Nitric oxide affects seed physiology by counteracting ABA signalling and dormancy maintenance, thereby promoting germination (Arc et al., 2013; Nagel et al., 2019) and in Arabidopsis, NO levels can be regulated by the extranuclear-localized NAC-domain TF, ANAC089 (Albertos et al., 2021). Members of the NAC gene family have been suggested to play important roles in the regulation of the transcriptional reprogramming associated with plant stress response (Singh et al., 2021; Alshareef et al., 2022). For example, in rice NAC domain-containing proteins can interact with phytohormones and regulate plant response to different stress factors, i.e. by modulating ABA signalling (Hong et al., 2016). ANAC089 can bind the specific *cis*-regulatory CGTnAG motif that is overrepresented in the promoters of downregulated ABA-responsive genes and of upregulated redox-related homeostasis genes, and Arabidopsis seeds of the *gap1* mutant with a gain-of-function mutation in *ANAC089* showed increased tolerance against abiotic stress factors (Albertos et al., 2021). Changes in the abundance of the TFs ABI5, ANAC089 and of the stress marker, MDA, were assessed in combination with in-depth plant and seed phenotyping in the two barley genotypes, and metabolism of the embryo was evaluated by GC-MS-based metabolite profiling.

## Materials and methods

### Plant material, growth conditions and phenotyping

Caryopses (in which seed and fruit coats are fused), hereafter termed “seeds” for simplicity, of two barley genotypes (*Hordeum vulgare* L. convar. *distichon* (L.) Alef. var. *nutans* (Rode) Alef.), the Austrian landrace HOR 2110 (https://doi.org/10.25642/IPK/GBIS/18216 termed “B1”) and the breeding line HOR 4710 (https://doi.org/10.25642/IPK/GBIS/94164 termed “B2”) originally received from the VEG Saatzucht Boldebuck/Güstrow, were taken after 40 years of long-term storage at 0°C at the Federal *ex situ* Genebank for agricultural and horticultural plants of Germany (IPK Gatersleben). Seeds were multiplied in 2012 and the progeny was used for experiments in 2014. Of each genotype, 336 seedlings were grown in a greenhouse in pots (30 x 30 x 15 cm, 4 plants per pot) at a 23/15°C day/night cycle until anthesis. After half of the spikes had flowered, plants were kept for another seven days under the same conditions, and then randomly selected and subjected either to “control” conditions (C, 22/18°C and regular watering) or “elevated temperature” (ET, 28/25°C and regular watering), or “drought” (D, 22/18°C and 15% field capacity). After harvest, seeds were cleaned, dried at 20°C and 20% relative humidity (RH) for eight weeks (for after-ripening), and then stored at -18°C. The agronomic traits days to anthesis, days to maturity, number of spikes on the tillers and of seeds harvested, plant height, spike length, thousand seed weight (TSW) and seed grades (<2.2 mm, >2.2 mm, >2.5 mm and >2.8 mm) were evaluated.

### Seed phenotyping: Germination, vigour test, stratification and controlled deterioration

Three replicates of 40 seeds were placed on moistened filter paper and germinated at 5, 10, 20 or 25°C under 150 μmol m^-2^ s^-1^ for 8 h light/16 h dark cycle. Total germination (TG) was scored daily and considered completed when the radicle had protruded by more than 2 mm. Root and shoot lengths of seedlings were recorded for untreated (non-stratified) seed lots. After 14 days, seeds were categorised for vigour testing into normal seedlings (NS), abnormal seedlings (damaged, deformed, or decayed with no potential to develop into a normal plant), and non-germinating seeds according to ISTA (2014). For germination in the presence of ABA, seed germination and germination speed was tested according to Collin et al. (2020). For this, 35 seeds were placed in a Petri dish containing two layers of filter papers wetted with 12 mL distilled water (control) or ABA solution (75 and 300 μM). After a four-day stratification treatment at 4°C, seeds were germinated in a growth chamber at 22°C under an 8 h light/16 h dark cycle, and germination was scored daily. Total germination, the percentage of NS, the time to reach 10% and 50% of maximum TG (T_10_, T_50_), and the area under the germination progress curve (“area under curve”; AUC) after 100 h were calculated using the curve-fitting module of the Germinator software (Joosen et al., 2010). Cold stratification was conducted at 10°C for 7 days before transfer to 20°C for another week. For controlled deterioration, seeds were equilibrated at 24°C and 75% RH above NaCl for 10 days, and then subjected to 40°C for up to 9 weeks, during which time they lost viability, assessed by their failure to germinate.

### Determination of seed reserves

Seed protein, lipid and starch contents were extracted from freeze-dried and finely ground seed material. Total nitrogen content was measured by elemental analysis using a Vario EL3 (Elementaranalysesysteme GmbH) and multiplied by a known conversion factor, 5.7, for wheat grains (Sosulski and Imafidon, 1990), to estimate the total protein content. Total lipid content was analysed using nuclear magnetic resonance spectroscopy (NMR; MQ-60, Bruker GmbH) according to the manufacturer’s instructions. Starch was determined using near infrared (NIR) spectroscopy (MPA, Multi Purpose Analyzer, Bruker GmbH), applying multivariate calibration algorithms (software OPUS, Bruker GmbH) and the reference material library B-FING-S (Bruker GmbH).

### Assessment of malon dialdehyde and transcription factors

Malondialdehyde was determined as described in Bailly and Kranner (2011). Seeds were ground using a chilled mortar and pestle with 5 mL of thiobarbituric acid:trichloroacetic acid solution of 0.5% (w/v):20% (w/v), the homogenate was heated to 95°C and kept for 30 min in a water bath and then quickly cooled on ice. After centrifugation of the homogenate at 16,000×*g* for 30 min, 2 mL of the supernatant was used for MDA determination. Equivalents of MDA were calculated from the difference in absorbance at 532 and 600 nm using an extinction coefficient of 155 mM^-1^ cm^-1^.

For the assessment of ABI5 and NAC-domain-containing TFs, total proteins were extracted from dry seeds or after imbibition in sterile distilled water for 12 h in the dark. Seeds were ground using mortar and pestle and homogenized in extraction buffer (100 mM Tris-HCl, 150 mM NaCl, 0.25% NP-40; v/v) containing 1 mM PMSF and 1 x cOmplete^®^ EDTA-free protease inhibitors (Sigma) followed by centrifugation for 10 min at 15,800 g at 4°C. Final protein concentration was determined by the Bio-Rad Protein Assay (Bio-Rad) based on the Bradford method (Bradford, 1976). In total, 45 µg of total protein per well were loaded in SDS-acrylamide/bisacrylamide gel electrophoresis using Tris-glycine-SDS buffer. Proteins were electrophoretically transferred to a 0.2 µM polyvinylidene difluoride (PVDF) membrane (Bio-Rad) using the Trans-Blot Turbo (Bio-Rad). Membranes were blocked in Tris-buffered saline-0.1% Tween 20 containing 5% blocking agent and probed with antibodies diluted in blocking buffer with 1% blocking agent. Anti-ANAC089 purified rabbit immunoglobulin (Biomedal, 1:10,000), a polyclonal antibody binding to the Arabidopsis ANAC089 (Albertos et al., 2021), and anti-ABI5 purified rabbit immunoglobulin (Biomedal, 1:10,000) (Albertos et al., 2015), anti-Actin (Sigma, 1:10,000) and ECL-peroxidase-labelled anti-rabbit (Amersham, 1:10,000) and anti-mouse (Amersham, 1:10,000) antibodies were used in the western blot analyses. Proteins were detected using ECL Advance Western Blotting Detection Kit (Amersham) by their chemiluminescence using a ChemiDoc MP Imaging System (Bio-Rad).

### Metabolite profiling

For metabolite profiling, three replicates of 20 mg of barley embryos manually dissected from dry mature seeds were ground with mortar and pestle in liquid nitrogen and stored at -80°C. Each sample was placed in a 2 mL Safe-Lock Eppendorf tube (Eppendorf AG). The ground, frozen samples were re-suspended in 1 mL of ice-cold (-20°C) water:acetonitrile:isopropanol (2:3:3 v/v/v) containing 4 mg L^-1^ ribitol as an internal standard and extracted for 10 min at 4°C by shaking (1,400 rpm) with an Eppendorf Thermomixer (Eppendorf AG). Insoluble material was removed by centrifugation at 20,000 g for 5 min. 25 µL were collected and dried for 150 min in a SpeedVac (Savant SPD131DDA, Thermo Fisher Scientifi) and stored at -80°C. Seed samples were removed from the freezer and allowed to equilibrate with ambient temperature for 15 min before opening and drying in a SpeedVac for 1 hour before adding 10 µL of 20 g L^-1^ methoxyamine in pyridine to the samples, the blanks or the standard solutions of amino acids and sugars. After 90 min of continuous shaking in an Eppendorf Thermomixer at 28°C, 90 µL of N-methyl-N-trimethylsilyl-trifluoroacetamide (MSTFA) were added and the reaction continued for 30 min at 37°C. After cooling, 50 µL were transferred to an autosampler vial. Two hours after derivatization, 1 µL of sample was injected in splitless mode into an Agilent 7890A gas chromatograph coupled to an Agilent 5975C mass spectrometer and separated on a Rxi-5SilMS column (Restek, 30 m with 10 m Integra-Guard column). The liner (Restek # 20994) was changed before each series of analysis and 10 cm of column were cut. Oven temperature ramp was 70°C for 7 min, then 10°C min^-1^ to 325°C for 4 min for a total run time of 36.5 min. Helium flow was 1.5231 mL min^-1^, injector temperature 250°C, transfer line temperature 290°C, ion source temperature 250°C and quadrupole temperature was 150°C. Samples and blanks were analysed in a randomized manner. Amino acid standards were injected at the beginning and end of each sample batch for monitoring of the derivatization stability. An alkane mix (C10, C12, C15, C19, C22, C28, C32, C36) was injected in the middle of the queue for external calibration of retention indices. Five scans per second were acquired. Raw Agilent data files were converted to NetCDF format and analysed with AMDIS (http://chemdata.nist.gov/mass-spc/amdis/), and metabolites identified according to their retention indices and mass spectra. Peak areas were then analysed using the QuanLynx software (Waters Corporation, Milfords, MA, USA) after conversion of the NetCDF file to MassLynx (Waters) format. Two analytical replicates of each biological replicate (n=3) were analysed. The data is available as part of the Metabolomics Workbench repository (Project: R001453; datatrack_id:3436, study_id:ST002272; (Sud et al., 2016) under http://dx.doi.org/10.21228/M8X13R.

### Statistics

Data for plant and seed phenotyping and for seed reserves were tested for normality using the Shapiro-Wilk test. If no normal distribution was found, the Kruskal Wallis one-way analysis of variance of ranks was used. Then, data were tested for significance (P<0.05) by the Tukey or Dunn’s *post-hoc* test, as appropriate. For the metabolite profiling data, raw values were transformed *via* log1p transformation (formula = ln(value + 1)) and imputation of missing values was performed using the R package ‘missForest’ (Stekhoven and Bühlmann, 2011). Metabolites displaying more than 20% of missing values were not considered for the statistical analysis. To test for significances in the metabolite data set, two-way ANOVA and Benjamini and Hochberg false discovery rate (FDR) adjustment were used at P<0.05. Multivariate statistical analysis was performed using Principal Component Analysis (PCA) to assess differences between metabolites profiles for the six groups (2 genotypes x 3 growth conditions).

## Results

### Effects of the maternal environment on plant and seed phenotype

**Figure 1:**
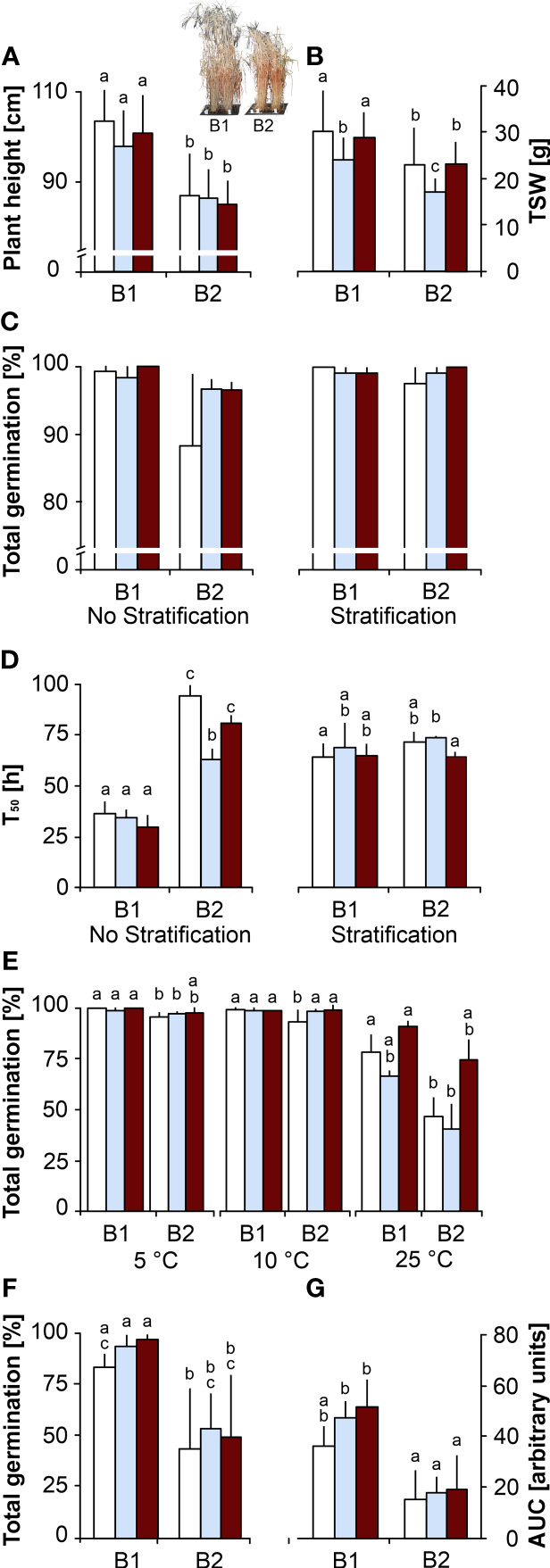
Effects of the maternal environment on plant and seed phenotype. Plants of genotypes B1 and B2 were grown in a greenhouse set to a 23/15°C day/night cycle until anthesis and then subjected to control conditions (22/18°C and regular watering; white bars), drought stress (22/18°C and 15% field capacity; blue bars) or elevated temperature (28/25°C and regular watering; red bars). Seeds were germinated at 20°C under an 8 h light/16 h dark cycle. The effects of the maternal environment are shown for **(A)** plant length (the inset shows a typical picture of the B1 and B2 plant phenotypes), **(B)** thousand seed weight (TSW), **(C, D)** total germination and time to reach 50% total germination (T_50_), respectively, with and without breaking of dormancy after stratification by exposing imbibed seeds to 10°C for 7 days, **(E)** total germination at 5, 10 and 25°C, **(F, G)** total germination and time to reach 10% total germination (T_10_), respectively, after 4 weeks of controlled deterioration at 75% RH and 40°C. Bars labelled with the same or no letters do not differ significantly at P<0.05; bars show mean ± SD of n = 112 plants in **(A, B)** and n = 3 x 40 seeds each in **(C-F)**.

**Controls.** The barley genotype B1 developed significantly taller plants (**Figure 1A**) that flowered earlier (**Figure S1A**) and had longer spikes (**Figure S1D**) producing heavier (**Figure 1B**) and larger seeds (**Figure S1F**) than B2. When germinated without stratification, TG and % NS of B2 seeds were lower than in B1 seeds (**Figure 1C**; **Figure S2A**) and this difference became significant when viewed through T_50_ (**Figure 1D**), revealing that B1 seeds germinated faster and produced longer shoots (**Figure S2B**). Differences in TG, % NS and T_50_ were mitigated when seeds were stratified prior to germination at 20°C (**Figures 1C, D**; **Figure S2A**). The significantly lower TG of B2 seeds when germinated at 25°C, but only to a small degree at 5 or 10°C (**Figure 1E**), confirmed that B2 seeds exhibited a more pronounced thermodormancy, which was broken by the stratification treatment (**Figure 1D**). When seeds were germinated in the presence of ABA, TG of seeds of both genotypes dropped significantly with increasing ABA concentration, with a 40% decrease in response to 300 μM ABA (**Figure S2E**). At this concentration, T_50_ of B1 was significantly delayed compared to B2 (**Figure S2E**). In response to artificial ageing by CD, used as a vigour parameter, seeds of both genotypes lost TG after 9 weeks of CD (**Figure S2C, D**). However, B1 seeds resisted the ageing treatment longer than B2 seeds, as shown by significantly compromised TG after 3 and 5 weeks of CD for B2 and B1 seeds, respectively (**Figure S2C, D**). Germination speed, expressed as AUC, was halved after 4 weeks of CD (**Figure 1G**).

**Drought.** Compared to controls, plants exposed to drought upon anthesis had significantly reduced spike numbers (**Figure S1C**), and their seeds needed less time to mature (**Figure S1B**), were lighter (**Figure 1B**) and smaller (**Figure S1F**) in both genotypes. Drought in the maternal environment did not affect TG in genotype B1, but the thermodormancy observed in B2 control seeds was reduced when seeds were produced under drought, which showed higher TG (**Figure 1C**) and faster germination at 20°C without stratification (**Figure 1D**; P<0.05). The effects of increasing ABA concentration on TG and T50 were less pronounced in seeds produced under drought in both genotypes, although B1 seeds took more time to germinate than B2 seeds (**Figures S2E, F**). In both genotypes, seeds produced under drought tended to resist CD slightly better than control seeds, but this effect was not significant at P<0.05 (**Figures 1F, G**; **Figures S2C, D**).

**Elevated temperature**. Compared to controls, plants grown under elevated temperature had significantly reduced spike numbers and seeds needed less time to mature in genotype B1 only (**Figures S1B, C**); TSW was not affected in either genotype (**Figure 1B**). The thermodormancy observed in B2 control seeds was reduced in seeds produced under elevated temperature (**Figures 1C-E**), which tended to resist CD slightly better than control seeds (not significant at P<0.05; **Figures S2C, D**). Compared to control seeds, seeds produced under elevated temperature and germinated in the presence of ABA had higher TG and germinated faster (lower T50), especially at 75 mM ABA (**Figure S2E, F**).

In summary, plants of genotype B1 were higher, flowered earlier and produced heavier, larger and more vigorous seeds without thermodormancy that germinated better in response to ABA and resisted ageing longer than those of genotype B2. Drought during seed development significantly reduced seed weight and size, and therefore total seed yield, in both genotypes, and elevated temperature reduced seed size, but not TSW significantly. Drought and elevated temperature in the maternal environment reduced thermodormancy and affected germination in response to ABA in genotype B2. Seeds produced under drought and elevated temperature in the maternal environment tended to resist CD slightly better than control seeds.

In addition, we show a re-analysed subset of phenotyping data for the B1 and the B2 genotypes grown in four different years in the field (Nagel et al., 2019; Tarawneh et al., 2020), showing that some traits of B2, such as shorter plant height (**Figure S3D**) and dormancy (**Figure S3H-J**) were also expressed in the field, but other traits were improved compared to the plants grown in the greenhouse in B2 (**Figure 1**, **Figure S1**); for example, genotype B2 and genotype B1 had the same or better performance in the field regarding TSW (**Figure S3F**) and harvest index (**Figure S3G**), respectively.

### Effects of the maternal environment on seed reserves, malon dialdehyde and transcription factors

**Figure 2:**
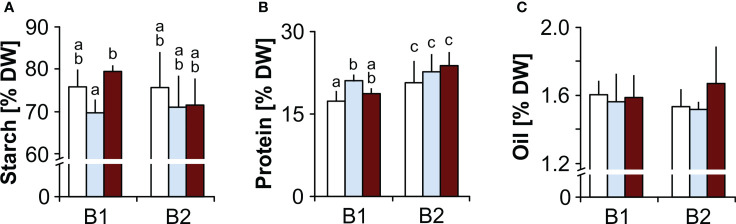
Effects of the maternal environment on seed reserves. Plants of genotypes B1 and B2 were grown in a greenhouse set to a 23/15°C day/night cycle until anthesis and then subjected to control conditions (22/18°C and regular watering; white bars), drought stress (22/18°C and 15% field capacity; blue bars) or elevated temperature (28/25°C and regular watering; red bars). The effects of the maternal environment on **(A)** starch, **(B)** protein and **(C)** seed oil contents are shown. Within each genotype, bars labelled with the same or no small letters do not differ significantly at P<0.05; bars show mean ± SD of n = 3 x 40 seeds.

**Figure 3:**
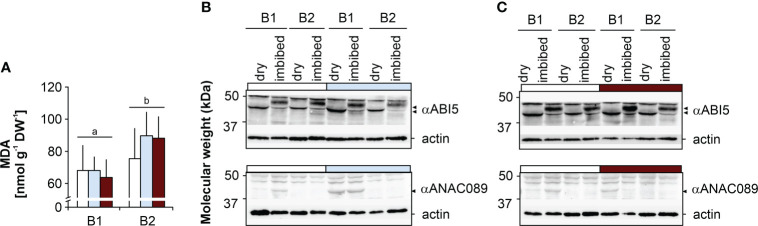
Effects of the maternal environment on malon dealdehyde (MDA) and the ABI5 and ANAC089-like transcription factors. Plants of genotypes B1 and B2 were grown in a greenhouse set to a 23/15°C day/night cycle until anthesis and then subjected to control conditions (22/18°C and regular watering; white bars), drought stress (22/18°C and 15% field capacity; blue bars) or elevated temperature (28/25°C and regular watering; red bars). The effects of the maternal environment are shown for **(A)** MDA (bars labelled with the same or no letters do not differ significantly at P<0.05; bars show mean ± SD of n = 3 x 40 seeds) and **(B)** the abundance of two transcription factors, ABI5 and ANAC089-like, in dry seeds and germinating seeds 12 h after the onset of imbibition after drought and **(C)** elevated temperatures.

**Starch, protein, oil and MDA contents.** Seeds of genotype B2 had significantly higher protein contents than B1 seeds, but comparable oil and starch contents. B1 seeds produced under drought had lower starch contents than seeds produced under elevated temperature, and compared to controls, B1 seeds had higher protein levels when produced under drought (**Figure 2**). Seed MDA levels were significantly higher in B2 seeds, and were not affected by the maternal environment in either genotype (**Figure 3A**).

**ABI5 and NAC domain-containing protein.** Dry seeds of both genotypes showed comparable ABI5 levels under control conditions (**Figures 3B, C**). In dry B1 seeds produced under drought (**Figure 3B**) and elevated temperature (**Figure 3C**), ABI5 levels appeared to be higher compared to control seeds. In imbibed seeds of both genotypes, a second ABI5 band with a slightly higher molecular weight was detected and up-accumulated as compared to dry seeds. Moreover, ABI5 levels were higher in B1 seeds produced under drought and under elevated temperature compared to B2 seeds, consistent with the higher tolerance to the CD treatment (**Figures 1F, G**, **S2C, D**). A NAC domain-containing protein with a similar size to ANAC089 (>37 kDa) was more abundant under all conditions in B1 compared to B2 (**Figures 3B, C**), consistent with its ability to produce more and heavier seeds even under adverse conditions compared to genotype B2 (**Figure 1B**; **Figure S1F**).

### Effects of the maternal environment on seed metabolite profiles

**Figure 4:**
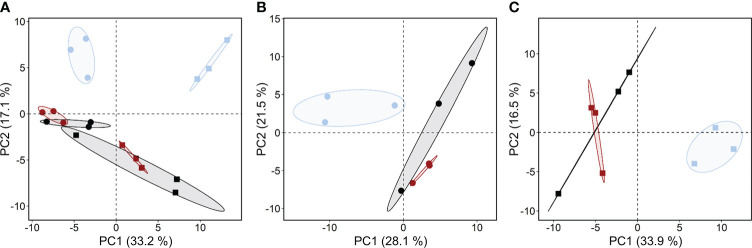
Effects of the maternal environment on metabolite profiles. Principal component (PC) analysis of all metabolites detected in embryos of barley seeds produced under different maternal environments. Plants of genotypes B1 (circles) and B2 (squares) were grown in a greenhouse set to a 23/15°C day/night cycle until anthesis and then subjected to control conditions (22/18°C and regular watering; black symbols), drought stress (22/18°C and 15% field capacity; blue symbols) or elevated temperature (28/25°C and regular watering; red symbols). The effects of the maternal environment on the metabolite profiles are shown for **(A)** both genotypes; **(B)** genotype B1 and **(C)** genotype B2.

**Principal component analysis of all metabolites across three maternal environments in genotypes B1 and B2.** In total, 173 compounds were detected and quantified (97 identified and 76 unknown molecules, **Table S1A**) in embryos isolated from dry barley seeds. After filtering for missing values, 140 metabolites (90 identified and 50 unknown metabolites, **Table S1B**) were considered for statistical evaluation. Visualization of the metabolite profiles by PCA revealed differences between the two genotypes along principal component (PC) 1, accounting for 33.2% of variance. In both genotypes, PC 2 clearly separated the metabolite profiles of embryos from seeds produced under drought, but not elevated temperature (**Figure 4A**). Separate PCA for each genotype (**Figures 4B, C**) confirmed that the effect of drought in the maternal environment on the metabolism of the embryo was more pronounced than that of elevated temperature.

**Figure 5:**
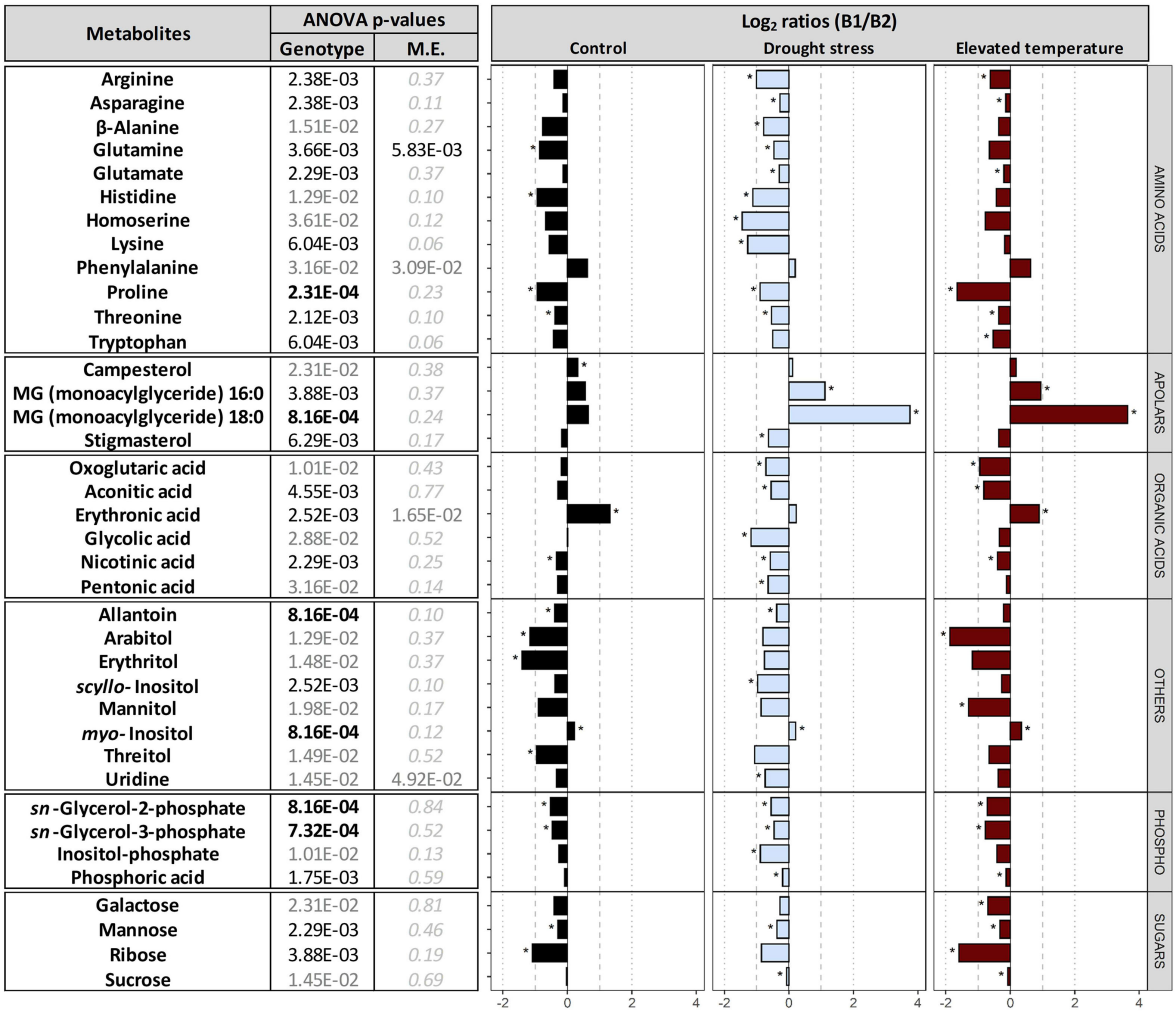
Differences in metabolites between genotypes. Plants of genotypes B1 and B2 were grown in a greenhouse set to a 23/15°C day/night cycle until anthesis and then subjected to control conditions (22/18°C and regular watering; black), drought stress (22/18°C and 15% field capacity; blue) or elevated temperature (28/25°C and regular watering; red). Metabolites differentially accumulated between the embryos of the two genotypes, across all maternal environments, were selected based on their two-way ANOVA p-value after false discovery rate (FDR) correction [left panel; bold black, black and grey numbers indicate significant differences at P<0.001, P<0.01 and P<0.05, respectively; light grey italic letters: not significant; interactive effects of genotypes x maternal environments (M.E.) were not significant]. The right panel shows log_2_ ratios for metabolites differentially accumulated between the two genotypes. Asterisks indicate significant differences between the two genotypes under a given M.E.

**Comparison between the two genotypes B1 and B2.** Comparison between the embryos dissected from dry seeds of the two genotypes revealed differential accumulation of 38 metabolites (**Figure 5**). In embryos of B2 seeds, the contents of most amino acids, organic acids, sugars, sugar alcohols and glycerol phosphates, were higher compared to B1 embryos in all three maternal environments. Specifically, under control conditions, B2 had higher levels of glutamine, histidine, proline, threonine, nicotinic acid, allantoin, arabitol, erythritol, threitol, sn-glycerol-2-phosphate and sn-glycerol-3-phosphate, mannose and ribose, whereas campesterol, erythronic acid and myo-inositol were significantly lower than in embryos of B1 seeds.

**Figure 6:**
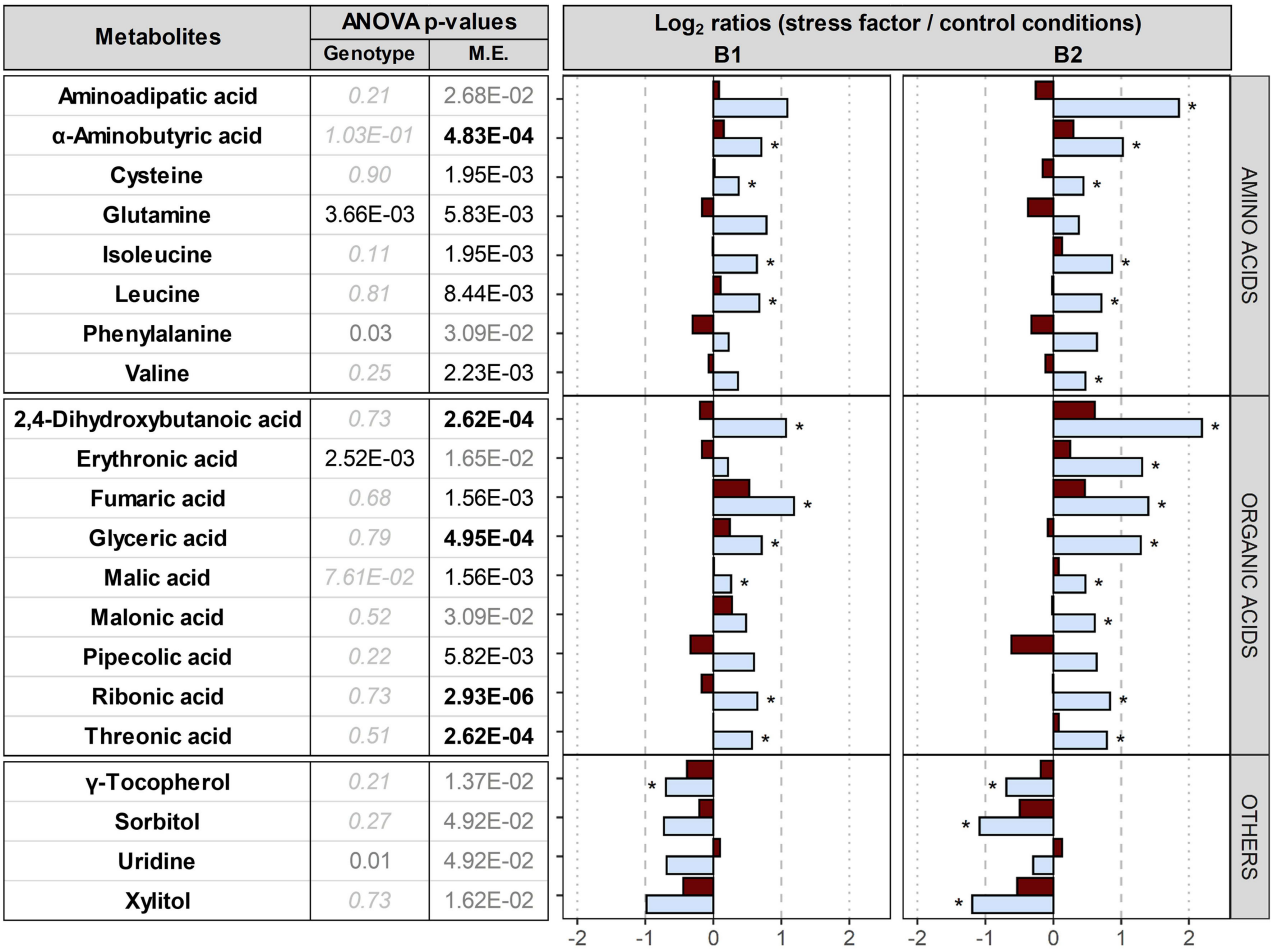
Impact of the maternal environment on metabolites. Plants of genotypes B1 and B2 were grown in a greenhouse set to a 23/15°C day/night cycle until anthesis and then subjected to control conditions (22/18°C and regular watering; black), drought stress (22/18°C and 15% field capacity; blue) or elevated temperature (28/25°C and regular watering; red). Metabolites differentially accumulated in embryos produced under the three maternal environments were selected based on their two-way ANOVA p-value after false discovery rate (FDR) correction [left panel; bold black, black and grey numbers indicate significant differences at P<0.001, P<0.01 and P<0.05, respectively; light grey italic letters: not significant; interactive effects of genotypes x maternal environments (M.E.) were not significant]. The right panel shows log_2_ ratios for metabolites differentially accumulated under drought or elevated temperature compared to control conditions. Asterisks indicate significant differences between drought or elevated temperature in the M.E. compared to control conditions.

**Comparison between control conditions and drought or elevated temperature.** The accumulation of 18 metabolites was differentially influenced by the maternal environment, especially by drought, leading to the up-accumulation of 8 amino acids, 9 organic acids and down-accumulation of γ-tocopherol, sorbitol, uridine and xylitol (**Figure 6**). No significant differences were found in embryos dissected from dry seeds produced under elevated temperature. In summary, drought, but not elevated temperature, upon seed maturation clearly affected the embryo metabolome, especially in B2.

## Discussion

We investigated if stress factors predicted to increase due to climate change, namely drought and elevated temperature, during seed production affect seed metabolism and seed quality traits in two genetically distinct genotypes of two-row spring barley, the Hana-type landrace B1 and the semi-dwarf B2. Plants of genotype B2 were significantly shorter, expressed more dormancy and had less vigorous seeds that deteriorated faster upon CD, and clear constitutive differences between genotypes in seed metabolism were identified (**Figures 1**, **5**). Semi-dwarf varieties carry mutation alleles related to changes in the metabolism and signal transduction of gibberellins or brassinosteroids (Dockter et al., 2014), and these phytohormones also affect seed traits. In seeds of the “EcoSeed” mapping panel, which included genotypes B1 and B2, the gibberellin 20-oxidases *HvGA20ox1* and *HvGA20ox3*, involved in the last steps of GA synthesis, were associated with pre-harvest sprouting and dormancy release (Nagel et al., 2019; **Figures S3H-J**). In addition, the genes *CONSTITUTIVE PHOTOMORPHOGENIC DWARF* (*HvCPD*), and *DIMINUTO* (*HvDIM*), both involved in brassinosteroid biosynthesis, and *HvBRI1*, which encodes a brassinosteroid receptor (Dockter et al., 2014), were also associated with dormancy (Nagel et al., 2019), and can also affect plant height (Dockter et al., 2014) and seed germination (Vriet et al., 2012). Plants with mutations in brassinosteroid synthesis or signalling pathways were also more susceptible to temperature stress, whereby hormonal interactions depended on genetic background and stress factor applied (Gruszka et al., 2016; Sadura et al., 2019). Taken together, the above studies provide evidence that traits selected for spring barley breeding involved modifications to the metabolism and/or signalling of gibberellins and brassinosteroids, which likely contributed to the differences between B1 and B2 in plant height and seed dormancy. However, germplasm selection for plant height can also introduce unwanted pleiotropic effects, such as late heading, low TKW, reduced seed size and yield and traits undesirable for brewing (Thomas et al., 1991). In field experiments, the B2 genotype did not show such compromised plant performance (Nagel et al., 2019; Tarawneh et al., 2020). However, later anthesis, lower TSW, reduced seed size and higher seed MDA levels in the B2 genotype were observed when plants were grown in the greenhouse (**Figures 1**, **3**; **Figure S1**), suggesting a lower tolerance to growth under sub-optimal environmental conditions, a trade-off sometimes found in semi-dwarfs (Thomas et al., 1991), i.e. genotype B2 appears to be more susceptible to stress than genotype B1.

Furthermore, the clear accumulation of a NAC domain-containing protein in dry and imbibed seeds of B1, but not B2, suggests that ABA metabolism and signalling are also differentially regulated in the two genotypes. NAC TFs have been reported to be involved in plant stress response generally, and in barley are implicated in the molecular crosstalk between ABA and jasmonic acid. Thus, the NAC gene family appears to be involved in regulatory pathways affecting agronomic traits (Christiansen et al., 2011). Genotype B1 accumulated more of a NAC-like orthologue, in agreement with the better resistance of B1 seeds to ageing, drought and elevated temperature (**Figures 1D, F, G**). As a polyclonal antibody against ANAC089 was used (Albertos et al., 2021), other NAC-like orthologues could have been detected, pointing at a similar NAC involved in stress responses of barley. On the other hand, seeds of the two genotypes had similar ABI5 levels under control conditions, but differed in dormancy status. The transcription factor ABI5, a key repressor of seed germination, regulates the expression of seed-specific genes (Finkelstein and Lynch, 2000) and is important for post-germination development (Lopez-Molina et al., 2001). The higher amount of ABI5 in response to drought and elevated temperature in the seeds of genotype B1, which have higher vigour and tolerate CD better, during imbibition also supports the view that B1 is more stress tolerant. Furthermore, ABI5 levels can remain unchanged while ABI5 function is altered. Besides NO-mediated degradation of ABI5 (Albertos et al., 2015), NO can reversibly modify thiol groups of specific cysteine residues (Stamler et al., 2001), affecting ABI5 redox state. B1 seeds with higher NO levels under field conditions compared to B2 were non-dormant (**Figure S3**), consistent with the previously reported correlation between NO release and lack of dormancy in barley (Nagel et al., 2019). Hence, subtle differences in the intricate network comprising hormonal interaction, *S*-nitrosylation and redox-regulation may account for differences in NO and ABA metabolism and signalling between the two genotypes, which may contribute to the different expression of dormancy and stress response in the two genotypes.

### Metabolite profiles reveal constitutive differences between the two genotypes

Metabolite profiling revealed clear differences between the embryos of the two genotypes, irrespective of the maternal environment under which the seeds were produced (**Figure 5**). The significantly higher abundance of 11 amino acids in B2 (**Figure 5**) is consistent with higher protein levels found in B2 seeds (**Figure 2B**). Higher levels of glutamine and amino acids synthesized from it, including arginine and proline (Forde and Lea, 2007), all of which were significantly enhanced in B2, support the synthesis of hordeins, which make up 30-50% of the barley proteins (Howard et al., 1996). This also agrees with the finding that levels of glutamic acid and proline were associated with high protein contents in barley seeds (Smith, 1972). Furthermore, accumulation of osmolytes and compatible solutes such as proline in response to various stress factors has been associated with antioxidative defence, signalling and chelation of metals (Hayat et al., 2012) and in spring barley, a protective role for proline in response to water shortage during spike formation was suggested to support seed development (Frimpong et al., 2021). Other compatible solutes, including sugar alcohols, such as arabitol, erythritol, mannitol and threitol, were also more abundant in B2 than in B1, regardless of the maternal environment, and indicative of an elevated stress response of the B2 genotype compared to B1.

In Arabidopsis, histidine was shown to promote oil deposition by activating genes of ABA biosynthesis (Ma and Wang, 2016). Therefore, histidine accumulation in B2 (**Figure 5**) leading to higher ABA contents would be consistent with the higher dormancy levels in B2 seeds (**Figures 1C, D**). Although the total lipid contents in seeds did not differ between the two genotypes (**Figure 2**), the differential accumulation of two monoacylglycerols and glycerol phosphates in isolated embryos (**Figure 5**) could also point at differences in lipid metabolism, possibly linked to lipid composition or distribution between endosperm and embryo. Furthermore, an accumulation of glycerol-2-P and glycerol-3-P has been associated with lower seed germinability after storage (Wiebach et al., 2020), in agreement with the lowered resistance to CD and increased level of MDA of B2 seeds.

Campesterol, a key precursor of brassinolide (Grove et al., 1979), was up-accumulated in embryos of the B1 genotype (**Figure 5**). Brassinosteroids promote germination by activating GA and inactivating ABA signalling (Kim et al., 2019), and a role for brassinolide in promoting plant growth and stress resistance through down-regulation of ABA signalling has been suggested (Bajguz and Hayat, 2009). Although an elevated level of a phytohormone precursor alone does not allow one to draw conclusions on signalling, the higher levels of campesterol in B1 embryos are at least consistent with the lack of dormancy in B1 seeds and their enhanced resistance to CD. Furthermore, *myo*-inositol was also significantly up-accumulated in B1 seeds, and this could be related to ABI5, which down-regulates PHOSPHATE1 (PHO1) gene expression associated with phosphate homeostasis and phosphorus mobilization from phytic acid (Wild et al., 2016; Huang et al., 2017), and variations in the *myo-inositol monophosphatase (CaIMP)* gene was associated with increased seed size in chickpea (Dwivedi et al., 2017). Therefore, ABI5 may control the phytate pool *via PHO1* and modulate *myo*-inositol metabolism, supporting increased seed size and lower stress levels in genotype B1. In summary, differences in the metabolite profiles in the embryos of the two genotypes produced in different maternal environments reflect clear genetic differentiation, also accounting for some of their individual traits.

### Effects of drought and elevated temperature in the maternal environment on metabolites

When elevated temperature was applied during seed filling, TSW, a key yield parameter, was unaffected in both genotypes. Genotype B1 matured earlier and produced less spikes, and B1 seeds had less thermodormancy, but these effects were not reflected in the metabolites assessed in this study, none of which were significantly changed in seeds produced under elevated temperature compared to controls (**Figure 6**). However, B1 seeds produced under drought or elevated temperature had higher ABI5 levels, especially upon imbibition. In barley, *HvABI5* is involved in the ABA-dependent regulation and fine-tuning of plant response to drought stress, associated with better membrane protection, higher flavonoid contents, and faster stomatal closure (Collin et al., 2020). In Arabidopsis, ABI5 binds to the promoter regions of LEA genes, such as *AtEm1* and *AtEm6*, delaying LEA protein accumulation in ABI5 mutants (Carles et al., 2002). Mutants deficient in AtEm6 fail to develop normal seeds due to a lower capacity to buffer water loss, resulting in premature dehydration in the seed filling phase (Manfre et al., 2005). Following this line of reasoning, higher ABI5 levels in B1 seeds could have led to a better water buffering capacity, supporting the development of more vigorous seeds compared to those of the B2 genotype.

In contrast to elevated temperature, drought applied during seed filling clearly affected seed metabolites in the embryos of both genotypes, and the effects of drought were very similar in both genotypes, irrespective of their distinct genetic backgrounds. Drought during seed filling resulted in lower TSW in both genotypes compared to controls (**Figure 1**), and alleviated pre-harvest sprouting and dormancy in B2 seeds under field conditions (**Figures S3H–J**), in agreement with previous reports (Gualano and Benech-Arnold, 2009). Hong et al. (2020) also reported that drought applied in the seed filling phase reduces seed weight and alters ABA signalling, and suggested a key role for reactive oxygen species (ROS) and flavonoids in drought response of barley by comparing a drought-tolerant Tibetan crop wild relative, *H. spontaneum*, to a drought-sensitive *H. vulgare* genotype.

Drought during seed production led to up-accumulation of amines and amino acids (**Figure 6**); 2-aminobutyric acid, cysteine, isoleucine and leucin were up-accumulated in both genotypes and also aminoadipic acid and valine in B2, together with elevated seed protein contents (significant for B1). Branched-chain amino acids, i.e. leucin, valine and isoleucine (Galili et al., 2016), contribute to target of rapamycin (TOR) activation and signalling, an evolutionarily conserved hub of nutrient sensing and metabolic signalling. Of the two distinct multiprotein complexes of TOR, TORC1 is highly conserved in all eukaryotes, and is implicated in protein synthesis and cell proliferation (Cao et al., 2019). In maize and barley, drought enhances the expression of genes involved in N uptake and assimilation in roots, promoting amino acid accumulation (Chloupek et al., 2010; Wang et al., 2017). In addition, a key step in sulphur assimilation is the incorporation of sulphur taken up by plant roots into cysteine, which is a precursor for all other organic sulphur-containing molecules, including methionine and the antioxidant glutathione, and can be further catabolized to pyruvate (Hell et al., 2002). Therefore, the observed up-accumulation of amino acids (**Figure 6**) supports previous findings that drought during seed filling enhances nitrogen and sulphur assimilation, and/or deposition of these two elements into seeds relative to carbon.

Furthermore, organic acids and osmolytes such as erythronic, threonic, fumaric, glyceric, pipecolic and ribonic acids were up-accumulated in seeds produced under drought in both genotypes (**Figure 6**). Plant response to water deficit includes metabolic adjustment involving organic acids and osmolytes in all organs, e.g. in grape leaves or wheat seeds (Hochberg et al., 2013; Mahmood et al., 2020); glyceric acid was up-accumulated in wheat roots after drought exposure and may support water and nutrient uptake (Kang et al., 2019); pipecolic acid, a product of lysine catabolism, was also suggested to be involved in osmo-regulation in oilseed rape (Moulin et al., 2006). Erythronic and threonic acids were positively correlated with drought-induced yield reduction and were suggested to be markers of yield stability (Lawas et al., 2019). By contrast, sorbitol, xylitol and the lipophilic antioxidant γ-tocopherol, a member of the tocochromanols (the vitamin E family) found at high concentrations in barley embryo and scutellum (Falk et al., 2004; Abbasi et al., 2007), were down-accumulated in embryos of both genotypes in response to drought during seed production (**Figure 6**). γ-tocopherol has been suggested to effectively mediate osmoprotection by preventing oxidative damage to polyunsaturated fatty acids (Sattler et al., 2004; Abbasi et al., 2007), and the down-accumulation in barley embryos of both genotypes may indicate a relatively higher consumption for this antioxidant under drought. In summary, modifications to the accumulation of osmolytes, organic acids and γ-tocopherol in the embryos of both genotypes appears to reflect general metabolic re-arrangements in response to drought independent of genotype.

### Summary and conclusions

Traits selected for spring barley breeding, which led to the replacement of barley landraces, such as the Hana-type landrace B1, by semi-dwarf varieties such as genotype B2, involved modifications to phytohormone metabolism and/or signalling, including gibberellins, brassinosteroids and ABA. We do not intend to draw general conclusions about the metabolism of landraces *versus* semi-dwarfs, but our study points out that the particular semi-dwarf genotype B2, used here, also has trade-offs such as lower stress resistance, higher dormancy and higher seed nitrogen contents, compensated for by enhanced lodging resistance. Metabolite profiling revealed clear heritable differences between the two genotypes, irrespective of the maternal environment under which the seeds were produced, reflecting clear genetic differentiation. By contrast, drought affected seed metabolites in the embryos of both genotypes in a rather similar way, independent of genotype, with modifications to the metabolism of osmolytes, organic acids and γ-tocopherol, and enhanced nitrogen and sulphur assimilation. Our study may support future breeding efforts to produce new lodging and drought resistant malting barleys, by targeting re-introduction of some of the beneficial traits of old landraces such as lower dormancy and a generally higher stress resistance.

## Data availability statement

The original contributions presented in the study are included in the article/supplementary files. The GC-MS based metabolite profiling dataset was submitted to the Metabolomics Workbench repository (https://www.metabolomicsworkbench.org, datatrack_id:3436, study_id:ST002272). The DOI for this project (PR001453) is: http://dx.doi.org/10.21228/M8X13R. Further inquiries can be directed to the corresponding author.

## Author contributions

MN, LR, GCl, GCu, and MB conducted the main experiments together with CS, IS-V, and HR. MN, IK, EA, LR, GCl, GCu, CB, CS, TR, IS-V, HR, and OL analysed the data. MN and IK wrote the paper with inputs from all other authors. All authors contributed to the article and approved the submitted version.
