# Supplementary material for: Impacts of drought and elevated temperature on the seeds of malting barley
Source: Front Plant Sci. 2022 Dec 8;13:1049323. doi: 10.3389/fpls.2022.1049323 (PMC9773840; doi:10.3389/fpls.2022.1049323)
Supplement: Supplementary file 1 [file DataSheet_1.docx]

Supplementary Material

#

Impacts of drought and elevated temperature on the seeds of malting barley

**Manuela Nagel^1*^, Erwann Arc^2^, Loïc Rajjou^3^, Gwendal Cueff^3^, Marlene Bailly^3^, Gilles Clément^3^, Inmaculada Sanchez-Vicente^6^, Christophe Bailly^4^, Charlotte E. Seal^5^, Thomas Roach^2^, Hardy Rolletschek^1^, Óscar Lorenzo^6^, Andreas Börner^1++^, Ilse Kranner^2++^**

^1^Genebank Department, Leibniz Institute of Plant Genetics and Crop Plant Research (IPK), Corrensstraße 3, OT Gatersleben, 06466 Seeland, Germany; ^2^Department of Botany and Center for Molecular Biosciences Innsbruck (CMBI), University of Innsbruck, Sternwartestraße 15, A-6020 Innsbruck, Austria; ^3^Université Paris-Saclay, INRAE, AgroParisTech, Institut Jean-Pierre Bourgin (IJPB), 78000, Versailles, France; ^4^UMR 7622 Biologie du Développement, IBPS, Sorbonne Université, CNRS, F-75005 Paris, France; ^5^Royal Botanic Gardens, Kew, Wakehurst, Ardingly, Haywards Heath, West Sussex, UK; ^6^Department of Botany and Plant Physiology, Instituto de Investigación en Agrobiotecnología (CIALE), Facultad de Biología, Universidad de Salamanca, C/ Río Duero 12, 37185 Salamanca, Spain

*** Correspondence:** Nagel@ipk-gatersleben.de

**^++^joint last authors**

Manuela Nagel, ORCID <http://orcid.org/0000-0003-0396-0333>
Erwann Arc ORCID https://orcid.org/0000-0003-2344-1426
Loïc Rajjou ORCID http://orcid.org/0000-0001-9739-1041
Gwendal Cueff
Marlene Bailly
Gilles Clément
Inmaculada Sánchez -Vicente ORCID http://orcid.org/0000-0001-6608-5578
Christophe Bailly ORCID http://orcid.org/0000-0002-5878-9249
Charlotte E. Seal ORCID <http://orcid.org/0000-0002-9329-9325>
Thomas Roach ORCID https://orcid.org/0000-0002-0259-0468
Hardy Rolletschek ORCID http://orcid.org/0000-0002-8619-1391
Oscar Lorenzo ORCID http://orcid.org/0000-0001-9523-0789
Andreas Börner ORCID http://orcid.org/0000-0003-3301-9026
Ilse Kranner ORCID <https://orcid.org/0000-0003-4959-9109>

**Supplementary figures**

**
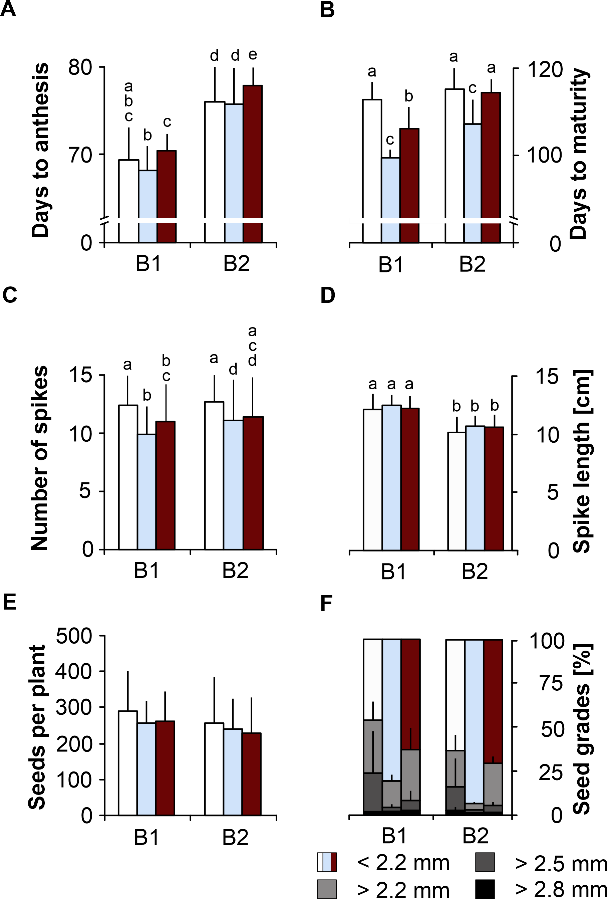
**

**Supplement Fig. S1. Effects of the maternal environment on the plant phenotype.** Plants of genotypes B1 and B2 were grown in a greenhouse set to a 23/15 °C day/night cycle until anthesis and then subjected to control conditions (22/18 °C and regular watering; white bars), drought stress (22/18 °C and 15 % field capacity; blue bars) or elevated temperature (28/25 °C and regular watering; red bars). Seed were germinated at 20 °C under an 8 h light / 16 h dark cycle. The effects of the maternal environment are shown for A) and B) days to anthesis and maturity, respectively, C) and D) spike number and length, respectively, E) seeds per plant and F) different grades of seed size from very large (>2.8 mm) to small (<2.2 mm). Bars labelled with the same or no letters do not differ significantly at P<0.05; bars show mean ± SD of n = 112 plants of n = 112 plants in A) to E) and n = 3 x 100 g seeds each in F).


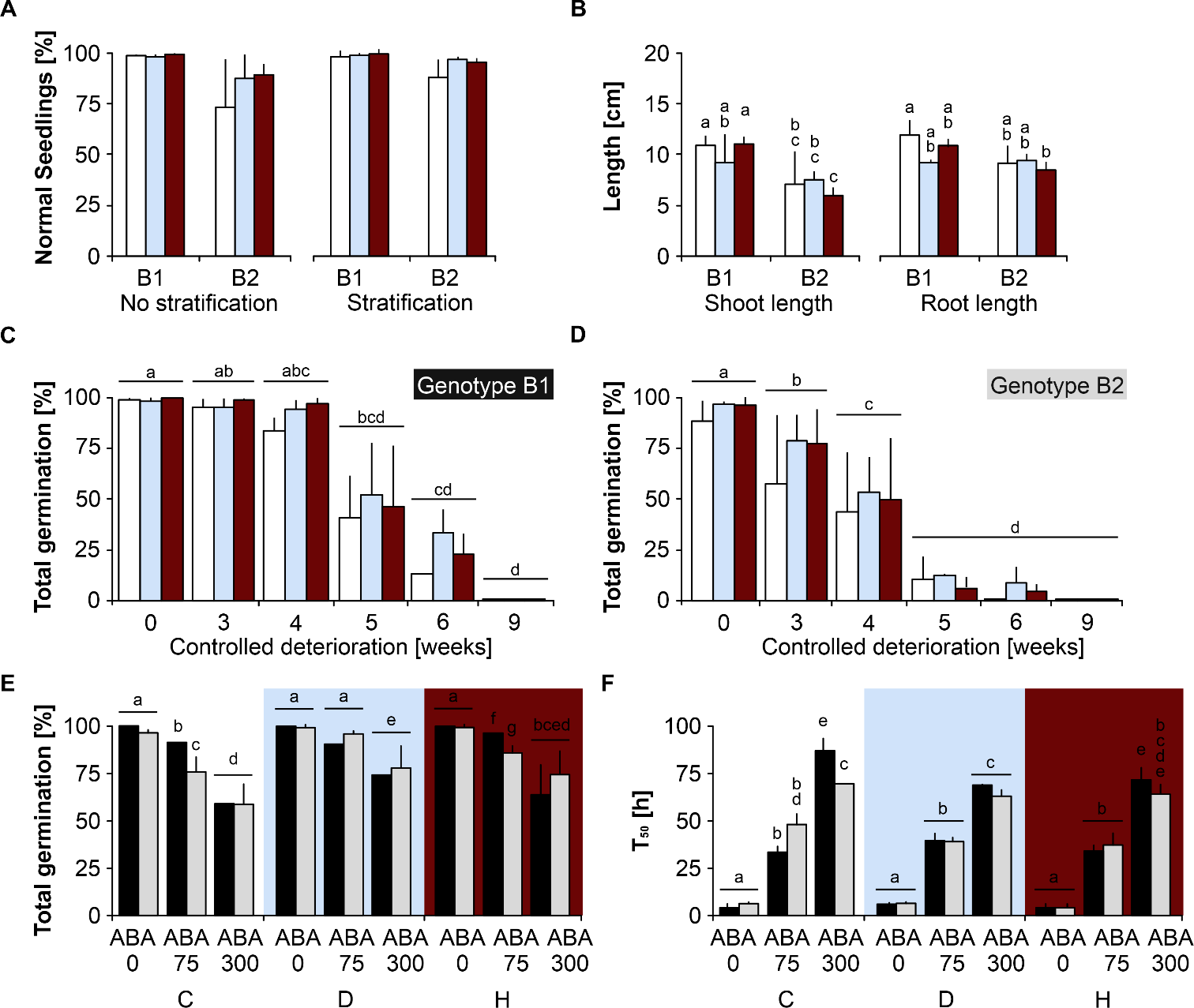


**Supplement Fig. S2. Effects of the maternal environment on seedlings.** Plants of genotypes B1 and B2 were grown in a greenhouse set to a 23/15 °C day/night cycle until anthesis and then subjected to control conditions (22/18 °C and regular watering; white bars), drought stress (22/18 °C and 15 % field capacity; blue bars) or elevated temperature (28/25 °C and regular watering; red bars). Seed were germinated at 20 °C under an 8 h light / 16 h dark cycle. The effects of the maternal environment are shown for **A)** normal seedlings germinated with and without breaking of dormancy by stratification using exposure of imbibed seeds to 10 °C for 7 days **B)** shoot and root length of normal seedlings without stratification. Total number of germinated seeds are shown for genotypes **C)** B1 and **D)** B2 after controlled deterioration at 75 % RH and 40 °C for up to 9 weeks. **E)** and **F)** total number of germinated seeds and time to reach 50 % total germination (T_50_), respectively is given for genotypes B1 (black bars) and B2 (grey bars) after germination of seeds using distilled water (0) or 75 μM (75) and 300 μM (300) abscisic acid (ABA) solution. Bars labelled with the same or no letters do not differ significantly at P<0.05; bars show mean ± SD of n = 3 x 40 seeds.

**
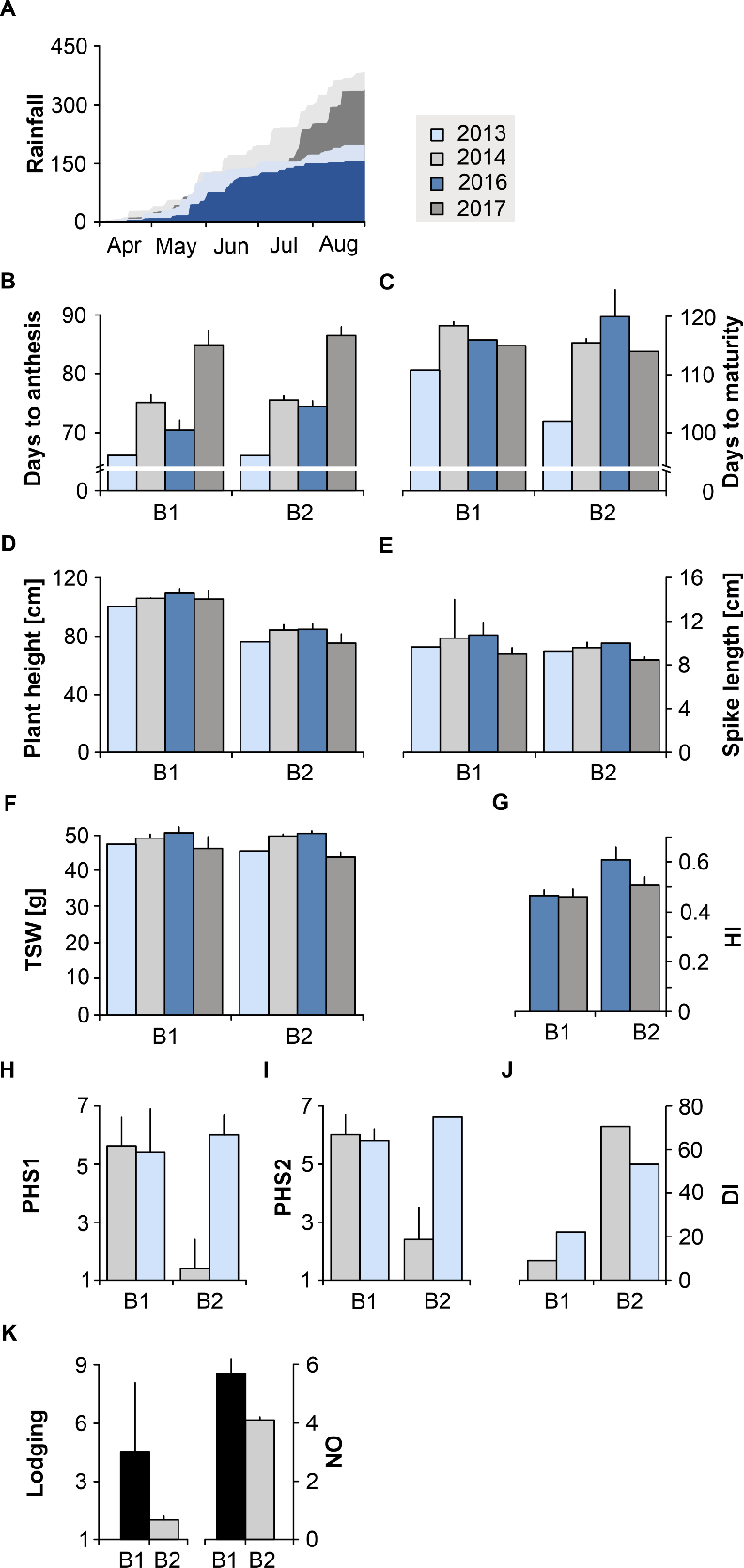
**

**Supplement Fig. S3: Growth and dormancy parameters of genotypes B1 and B2 grown in four different years.** Plants of genotypes B1 and B2 were grown as part of the EcoSeed mapping panel. Data for the years 2013 and 2014 published in Nagel et al. (2019) and for 2016 and 2017 in Tarawneh et al. (2020) were re-analysed and collated for ease of comparison between phenotypic traits of plants grown in the field (this figure) and plants grown in the greenhouse (Fig. 1, Figs. S1 and S2). **A)** Rainfall was measured daily at the Gatersleben estate and was twice as high in 2014 and 2017 compared to 2013 and 2016. Agronomic traits such as **B)** days to anthesis, **C)** days to maturity, **D)** plant height, **E)** spike length, **F)** thousand seed weight (TSW) are shown for all years. **G)** Harvest Index (HI) was analysed only in 2016 and 2017. In 2013 and 2014, **H)** preharvest sprouting (PHS) was evaluated in sand at maturity (PHS1) and I) 14 days after maturity (PHS2). **J)** Dormancy was assessed by dormancy index (DI) at maturity, when more dormant seeds had a higher DI. **K)** In 2014, lodging was evaluated between 1 (no lodging) and 9 (complete lodging) and nitric oxide release (NO) was estimated *via* its main autoxidation product, dinitrogen trioxide based on (Liu et al., 2016).

**References**

Liu, Y., Buerk, D.G., Barbee, K.A., and Jaron, D. (2016). A mathematical model for the role of N_2_O_3_ in enhancing nitric oxide bioavailability following nitrite infusion. *Nitric Oxide: Biology and Chemistry* 60**,** 1-9. <https://doi.org/10.1016/j.niox.2016.08.003>

Nagel, M., Alqudah, A.M., Bailly, M., Rajjou, L., Pistrick, S., Matzig, G., Börner, A., and Kranner, I. (2019). Novel loci and a role for nitric oxide for seed dormancy and pre-harvest sprouting in barley. *Plant Cell and Environment* 42**,** 1318-1327. <https://doi.org/10.1111/pce.13483>

Tarawneh, R.A., Alqudah, A.M., Nagel, M., and Börner, A. (2020). Genome-wide association mapping reveals putative candidate genes for drought tolerance in barley. *Environmental and Experimental Botany* 180**,** 104237. <https://doi.org/10.1016/j.envexpbot.2020.104237>
